# Single-isocenter stereotactic non-coplanar arc treatment of 200 patients with brain metastases: multileaf collimator size and setup uncertainties

**DOI:** 10.1007/s00066-021-01846-6

**Published:** 2021-09-15

**Authors:** Lucie Calmels, Susan Blak Nyrup Biancardo, Patrik Sibolt, Susanne Nørring Bekke, Ulf Bjelkengren, Eva Wilken, Poul Geertsen, David Sjöström, Claus F. Behrens

**Affiliations:** Department of Oncology, Radiotherapy Research Unit (52AA), Herlev & Gentofte Hospital, University of Copenhagen, Borgmester Ib Juuls vej 7, 2730 Herlev, Denmark

**Keywords:** Radiotherapy, Rotational errors, Geometric offset, Plan quality, IGRT, ExacTrac

## Abstract

**Purpose:**

The purpose of this study was to evaluate our 2 years’ experience with single-isocenter, non-coplanar, volumetric modulated arc therapy (VMAT) for brain metastasis (BM) stereotactic radiosurgery (SRS).

**Methods:**

A total of 202 patients treated with the VMAT SRS solution were analyzed retrospectively. Plan quality was assessed for 5 mm (120) and 2.5 mm (high-definition, HD) central leaf width multileaf collimators (MLCs). For BMs at varying distances from the plan isocenter, the geometric offset from the ideal position for two image-guided radiotherapy workflows was calculated. In the workflow with ExacTrac (BrainLAB, München, Germany; W‑ET), patient positioning errors were corrected at each couch rotation. In the workflow without ExacTrac (W-noET), only the initial patient setup correction was considered. The dose variation due to rotational errors was simulated for multiple-BM plans with the HD-MLC.

**Results:**

Plan conformity and quality assurance were equivalent for plans delivered with the two MLCs while the HD-MLC plans provided better healthy brain tissue (BmP) sparing. 95% of the BMs had residual intrafractional setup errors ≤ 2 mm for W‑ET and 68% for W‑noET. For small BM (≤1 cc) situated >3 cm from the plan isocenter, the dose received by 95% of the BM decreased in median (interquartile range) by 6.3% (2.8–8.8%) for a 1-degree rotational error.

**Conclusion:**

This study indicates that the HD-MLC is advantageous compared to the 120-MLC for sparing healthy brain tissue. When a 2-mm margin is applied, W‑noET is sufficient to ensure coverage of BM situated ≤ 3 cm of the plan isocenter, while for BM further away, W‑ET is recommended.

## Background

Brain metastases (BM) are the most common intracranial tumor and occur in 20–30% of patients with metastatic cancer [1]. The treatment options include surgical resection [2], whole-brain radiotherapy (WBRT) [3], and stereotactic radiosurgery (SRS) [4, 5].

Since its introduction, SRS has been increasingly employed for BM treatment and the technologies available have improved significantly. Introduction of the Gamma Knife (GK) device (Elekta AB, Stockholm, Sweden) [6] and more recently the CyberKnife (Accuray Inc., Sunnyvale, CA, USA) [7] as well as the linear accelerator (Linac)-based volumetric modulated arc therapy (VMAT) modalities has made it possible to deliver high-dose conformal radiation to multiple BM [8].

Improved multileaf collimator (MLC) design and higher dose rate and gantry speed, in conjunction with development of improved image-guided radiotherapy (IGRT) capabilities [9], have increased precision of the Linac-based VMAT solutions for patients requiring SRS. The first Linac-based VMAT SRS approach when treating multiple BM was typically one isocenter placed at each lesion, which was treated separately. This solution was challenging for treatment planning and gave rise to long overall treatment time (OTT) [10]. In order to shorten the treatment time and reduce adverse dosimetric effects related to intrafractional motion, multitarget single-isocenter non-coplanar VMAT SRS was proposed by different authors [10–12], proving equivalent dose distributions and overall survival compared to GK treatments [10]. The safe use of such a technique is dependent on extensive dosimetric validation and use of precise immobilization of the brain, combined with a six-degrees of freedom (6-DOF) alignment and an effective IGRT solution.

Commercial single-isocenter non-coplanar VMAT SRS solutions were recently developed to simplify the overall process, with dedicated optimization algorithms and support-device mask fixation [10, 12]. Investigations of these SRS treatment options have demonstrated dosimetric results comparable to GK and CyberKnife [10, 13–15].

In the present study, we report our 2 years’ experiences with a single-isocenter non-coplanar VMAT SRS solution utilized to treat multiple BM. The aim of this study was: 1) to evaluate two different MLCs by plan quality comparison and SRS quality assurance (QA), 2) to evaluate the setup accuracy and the intrafractional motion with different IGRT workflows, and 3) to estimate the risk of compromised target coverage due to residual isocenter rotational errors.

## Materials and methods

All single-isocenter non-coplanar VMAT SRS plans generated in this study were based on the HyperArc™ (HA; Varian Medical Systems Inc., Palo Alto, CA, USA) module within the Eclipse™ (v. 15.5.07, Varian Inc.) treatment planning system (TPS). The solution allows for SRS of single and multiple BM on TrueBeam (Varian Inc.) Linacs. The dedicated Encompass^TM^ SRS Immobilization (Qfix, Avondale, PA, USA) mask and support includes three markers (two laterally and one at the midline of the patient) that are aligned with the in-room lasers.

### Patients and simulation

This retrospective study included 202 patients (median age 67 years, range: 28–85 years; 90 males and 112 females) with 1–5 BM treated with the HA SRS solution between October 2018 and June 2020 at our institution. Patient characteristics are summarized in Table 1.

**Table 1:** Patient gender, patient median age (range), targets per plan, and median BM GTV volume

		120-MLC	HD-MLC
Number of patients	Male	14	76
	Female	18	94
	All patients	32	170
Median age (years, range)	All patients	67 (53–83)	68 (28–85)
Number of plans	1 BM	17	116
	2 BM	11	39
	3 BM	0	15
	4 BM	4	10
	5 BM	0	1
Median (IQR) GTV volume (cc)	All plans	1.7 (0.2–4.9)	1.3 (0.3–4.8)
	1 × 18 Gy	1.5 (0.2–4.6)	1 (0.2–3.3)
	3 × 9 Gy	56.2 (NA)	13.2 (6.3–19.1)
	1 × 13 Gy	10.5 (NA)	1.2 (0.2–3.2)

*IQR* interquartile range

The planning computed tomography (pCT) scan (Brilliance Big Bore, Philips, Amsterdam, Netherlands) was acquired in supine position (slice thickness of 1 mm, pixel matrix of 512 × 512 pixels, and field of view, FOV, of 37 cm, 120 kVp). A T_1_-weighted axial 3D (matrix = 560 × 560 pixels, FOV = 25 cm, slice thickness = 3.9 mm) and a T_2_-weighted axial TSE 2D (matrix = 512 × 512 pixels, FOV = 22 cm, slice thickness = 0.8 mm) magnetic resonance image (MRI) were registered to the pCT scan to support delineation of organ at risk (OAR) and the BM gross tumor volume (GTV) in Eclipse^TM^. An isotropic 2‑mm margin was added to the GTV to create the planning target volume (PTV).

### Treatment planning

All treatment plans were created for and delivered by a TrueBeam Linac utilizing a flattening filter-free beam with 10- or 6‑megavoltage (MV) photon beam quality at a maximum dose rate of 2400 monitor unit (MU)/min and 1400 MU/min, respectively. The Linac was equipped with a Varian Millenium^TM^ 120-leaf MLC (120-MLC; in each bank: 5‑ and 10-mm leaf width for the 40 central and the 20 outer leaves, respectively) for the first 32 patient treatments, whereas the rest of the patients were treated utilizing the Varian High Definition (HD) MLC (in each bank: 2.5- and 5‑mm leaf width for the 32 central and the 28 outer leaves, respectively).

The different dose fractionations were 3 fractions of 9 Gy for BM larger than 3 cm, 1 fraction of 13 Gy for BM close to one OAR (i.e., brainstem, chiasma), and 1 fraction of 18 Gy for the other BM.

Volumetric dose prescription was adopted, with plan normalization ensuring that the dose received by 98% of the total PTV (i.e., the union of all the PTVs, D_98%_) was equal to the prescribed dose (D_p_). An intratumor dose heterogeneity, D_2%_, of up to 150% of D_p_ was accepted. The maximum dose was constrained to below 13 Gy for the optic nerves, the chiasma, and the brainstem, and as close as possible to 0 Gy for the lens.

After delineation of the targets and OAR, the plan isocenter was automatically placed by the HA module at the center of mass of all the BMs or as close as possible to allow gantry rotation and was manually adjusted if needed (i.e., BM too far from the plan isocenter). The applied beam configuration used four arc fields (one with couch rotation of 0° and three non-coplanar arcs with couch rotations of 315°, 45°, and 90° or 270°) automatically arranged around the plan isocenter. For accurate modeling of the beam penumbra, which may influence the critical OAR dosimetry, adjustments were carefully carried out during commissioning of the SRS technique. Finetuning of the beam model was performed by varying the dosimetric leaf gap (range: 0.081 to 0.150 cm) in the algorithm to best fit the measured dose distribution by visual inspection of the dose gradient together with gamma evaluation. The Acuros XB (v 15.6.03, Varian Inc., Palo Alto, CA, USA) dose calculation algorithm was used, with a grid size and optimization resolution of 1.25 mm. During plan optimization, automatic lower dose objective (ALDO) as well as SRS normal tissue objective (NTO) were applied to ensure target coverage and reduce the dose delivered to healthy tissue.

Plan evaluation was accomplished through dosimetric indices, dose–volume metrics, and plan efficiency indicators. The dose distribution conformity to the shape and the size of the BM was evaluated with the conformity index (CI) [16, 17], where a value close to one indicated an acceptable plan quality. The dose fall-off outside the target, which is a predictor of complication due to the dose delivered to healthy brain tissue, was assessed by the gradient index (GI) as well as by the mean dose (Gy) and the absolute volume (cc) of healthy brain tissue (brain minus PTVs, BmP) receiving more than 12 Gy (V_12 Gy_). The homogeneity of the dose distribution within the BM volume was represented by the homogeneity index (HI) [17], where a value below 2 complies with the protocol. The modulation factor (MU/Gy), and the median overall treatment time (OTT) were recorded and evaluated.

The three indices were defined as follows:

1 $$HI=\frac{D_{\max }}{D_{p}}$$

2 $$CI=\left(\frac{V_{D_{p}}^{2}}{PTV\times PV}\right) $$

3 $$GI=\frac{V_{50\% }}{PV}$$

Where *D*_*max*_ denotes the maximum dose, PTV is the PTV volume (in cc), $V_{{D_{p}}}$the volume of the PTV which is covered by the *D*_*p*_, and PV the total volume covered by the *D*_*p*_ (cc).

### Quality assurance

Machine performance check (MPC^TM^, Varian Inc.) was carried out every morning and dosimetric patient-specific quality assurance (QA) was measured for each plan (results not reported in this paper) [18].

The Winston–Lutz test, used to evaluate the correspondence between the plan isocenter and the image radiation center was performed every morning by imaging the BrainLAB isocenter phantom with ExacTrac® (ET; BrainLAB, München, Germany), with the kilovoltage (kV) on-board imaging (OBI) system, and with the EPID imaging systems device with a SID of 150 cm [19, 20]. The flat phantom was placed on the treatment couch at a random location. Two kV-images were acquired at 0 and 270° and the center of the tungsten sphere was aligned with the reference images using the TrueBeam Treatment Console. Automatic couch shifts were used to move the phantom to the imaging isocenter. The position of the phantom was checked with the ET system and the phantom was irradiated with a 1.5 × 1.5-cm^2^ field onto the EPID at four gantry rotations (0°, 90°, 180°, and 270°, fields defined by the jaws), seven collimator rotations (each 45°, fields defined by the MLC with gantry and couch at 0‑degree rotation), and at four couch rotations (270°, 315°, 45°, and 90°, fields defined by the jaws). The acquired images were analyzed in DoseLab Pro (Mobius Medical Systems, LP, Houston, TX) software using its automatic Winston–Lutz analysis package to determine the offset between the center of the sphere and the radiation isocenter.

### Image guidance and intrafractional motion

**Fig. 1:**
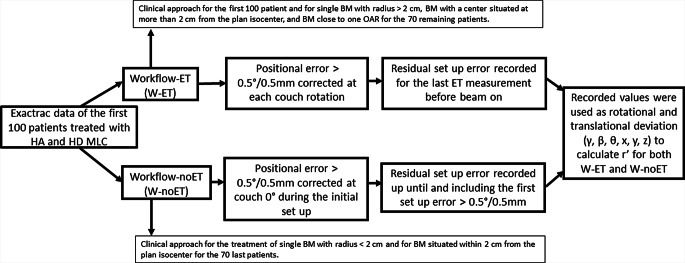
Diagram of the data recorded for W‑ET and W‑noET in order to calculate r′ and the maximum E‑curve deviation for each workflow

For the first 100 patients treated with HD-MLC, initial bony structure alignment correction was performed using cone-beam CT (CBCT) and the 6‑DOF couch. Then, prior to treatment of each arc, alignment was evaluated again using the ET system and, when necessary, corrections were made using the 6‑DOF couch, until the residual setup errors were within the fixed tolerance of 0.5º/0.5 mm. For these 100 patients, the distribution of residual intrafractional positioning errors was analyzed for the actually used workflow (W-ET) and for a simulated workflow (W-noET) that did not include ET corrections (Fig. 1). Analysis was done based on ET acquisition data (for each couch rotation). For W‑ET, the final residual error at each couch angle was recorded, i.e., the residual setup error as measured by the last acquired ET image set before beam on. For W‑noET, all residual setup errors measured by the ET system for each couch angle were recorded up until and including the first setup error that resulted in a 6-DOF couch correction. Residual setup errors after the first 6‑DOF couch adjustment were not included in the analysis for W‑noET. After analysis of setup errors for the first 100 patients treated with HD-MLC, the workflow was changed for the next 70 patients. W‑ET was used for single BM with radius >2 cm, BM with a center situated at more than 2 cm from the plan isocenter, and for BM close to one OAR. W‑noET was used for treatment of single BM with radius <2 cm and for BM situated within 2 cm from the plan isocenter.

The effect of rotational and translational setup errors on the position of a BM at a distance, r, from the plan isocenter was evaluated. The displacement of the BM center from the ideal position due to these setup errors was calculated according to the following geometric relationship [21]:

$$\begin{array}{cc} R_{\text{pitch}}\left(\theta \right)=\left(\begin{array}{cccc} 1 & 0 & 0 & 0\\ 0 & \cos \left(\theta \right) & -\sin \left(\theta \right) & 0\\ 0 & \sin \left(\theta \right) & \cos \left(\theta \right) & 0\\ 0 & 0 & 0 & 1 \end{array}\right) & R_{\mathrm{roll}}\left(\beta \right)=\left(\begin{array}{cccc} \cos \left(\beta \right) & 0 & \sin \left(\beta \right) & 0\\ 0 & 1 & 0 & 0\\ -\sin \left(\beta \right) & 0 & \cos \left(\beta \right) & 0\\ 0 & 0 & 0 & 1 \end{array}\right)\\ R_{\mathrm{yaw}}\left(\gamma \right)=\left(\begin{array}{cccc} \cos \left(\gamma \right) & -\sin \left(\gamma \right) & 0 & 0\\ \sin \left(\gamma \right) & \cos \left(\upgamma \right) & 0 & 0\\ 0 & 0 & 1 & 0\\ 0 & 0 & 0 & 1 \end{array}\right) & \mathrm{T}\left(x,y,z\right)=\left(\begin{array}{cccc} 1 & 0 & 0 & x\\ 0 & 1 & 0 & y\\ 0 & 0 & 1 & z\\ 0 & 0 & 0 & 1 \end{array}\right) \end{array}$$

$$\overline{r}=\left(rx\:ry\:rz\:1\right);\overline{r}'=R_{\text{pitch}}R_{\mathrm{roll}}R_{\mathrm{yaw}}Tr;E\left(r\right)=\left|\left|\overline{r}'-\overline{r}\right|\right|$$

where R_pitch_, R_roll_, R_yaw_ ($\text{pitch}\colon \theta ,roll\colon \beta ,\text{~and}\,yaw\colon \gamma$), and T (x: lateral, y: craniocaudal, z: anteroposterior) are the rotation and translation components, respectively. The distance between the center of the BM and the plan isocenter is denoted as $r=\left|\left|\overline{r}\right|\right|$. E is the magnitude of the displacement vector of the center of the BM from the ideal position.

The distances between the plan isocenter and the center of all lesions were recorded for each multi-BM treatment in order to determine the maximum value of r for our study.

The maximum values of E for a range of r values (from 0 to 8 cm, in 0.5 cm increments) were calculated to generate maximum E‑curves as function of r for W‑ET and W‑noET. For a given value of r, its coordinates were defined as $rx=ry=rz=\sqrt{\left(\frac{r^{2}}{3}\right)}$. In these calculations, to calculate $r'$, values for rotation deviations ($\theta ,\beta ,\upgamma$) and translation deviations $(x,y,z)$ were taken as certain percentiles from the recorded distribution of residual intrafractional positioning errors for W‑ET and W‑noET. First, all combinations of the 2.5th percentile (P_2.5_) and the 97.5th percentile (P_97.5_) were used and the combination giving the largest value of E was recorded. Since there were 6 DOF each with an upper and a lower percentile value, each recorded value for E requires 2^6^ = 64 calculations, where only the one yielding the highest E value was used. Second, all combinations of the 16th percentile (P_16_) and the 84th percentile (P_84_) were utilized in the same way. Thus, for each workflow, two maximum E‑curves were derived: one incorporating 68% of the recorded deviations (from P_16_ to P_84_) and another incorporating 95% of the data (from P_2.5_ to P_97.5_). These percentiles were chosen since the residual intrafractional positioning errors were not normally distributed (Lilliefors test) and 68 and 95% correspond approximately to 1 and 2 standard deviations for normally distributed data. To study the sensitivity of these curves, they were also calculated for six other r coordinate alternatives: three curves where either rx, ry, or rz equals r and the two others were set to zero, and three curves where either rx, ry, or rz were set to zero and the two others were equal to $\sqrt{\left(\frac{r^{2}}{2}\right)}$.

### Dosimetric effects of rotational errors

The dosimetric effects of the rotational errors were simulated using Velocity (v 4.0, Varian Medical System, Inc.) for the first multitarget plans with HD-MLC (*n* = 42). Rotations of ±0.5°, ±1.0°, and ±2.0° were applied uniformly about the three orthogonal axes from the plan isocenter. This was a simple rotation of the dose distribution relative to the CT, not an actual recalculation of dose. BM and OAR dose metrics were obtained by creating secondary dose–volume histograms (DVH).

The relative dose error between the reference plan and the rotated plan was calculated for the dose received by 95% of the GTV (D_95%_), for the D_max_ of the brainstem, and for the D_mean_ and the V_12_ _Gy_ of BmP. The data of D_95%_ received by the GTV after applying the rotational error was grouped according to BM size and distance above or below 3 cm from the isocenter.

### Statistical analysis

Statistical evaluation of the extracted parameters was performed in MATLAB (version R2019a, MathWorks, Natick, MA, USA). Lilliefors test was used to test whether a sample came from a normal distribution. Wilcoxon rank sum test was used to test whether two samples came from distributions with equal medians. The statistical significance level was chosen to be 5%. For the comparison between plans based on the two different MLCs, 22 tests were carried out and a Bonferroni correction for multiple testing was applied, giving a significance level of 5%/22 ≈ 0.23%. In all boxplots in this paper, the inner line denotes the median value, the box the interquartile range (IQR), the whiskers extend to the most extreme datapoints not considered outliers, and the outliers are presented as single markers. A datapoint is considered an outlier if its value is greater than $P_{75}+1.5\times \mathrm{IQR}$ or less than $P_{25}-1.5\times \mathrm{IQR}$. Notches indicate a 95% confidence interval around the median. Notches are calculated as $\mathrm{M}-1.57\times \frac{\mathrm{IQR}}{\sqrt{\mathrm{n}}}$ and $\mathrm{M}+1.57\times \frac{\mathrm{IQR}}{\sqrt{\mathrm{n}}}$, where *n* is the number of observations and M is the median value.

## Results

### HA treatment planning and delivery

Tables 2 and 3 summarize the data extracted from the plan calculated with HD-MLC and 120-MLC.

**Table 2:** Number of data and *p*-value summary of overall treatment time (OTT) with and without Exactrac (ET), and the modulation factor (MU/Gy) for the 3 fractionations schemes used. Data is for treatments utilizing HD-MLC

		Median (IQR)	Number of fraction or plan	*P*-value
*OTT (min) per fraction*	With ET	23 (20–31)	165	≤10^−23^
	Without ET	12 (11–13)	55	
*Modulation factor (MU/Gy) per plan*	Plan A: 1 × 18 Gy	339 (304–393)	145	A vs. B = 0.051
	Plan B: 3 × 9 Gy	320 (290–352)	25	A vs. C = 0.085
	Plan C: 1 × 13 Gy	367 (338–422)	12	B vs. C = 0.002

**Table 3:** Median (IQR) and *p*‑value summary of overall treatment time (OTT), D_mean_ and V_12Gy_ of BmP, HI, CI and GI index and modulation factor (*MU/Gy*). Data includes HA-plans utilizing both 120-MLC or HD-MLC

		120-MLC		HD-MLC		
		Median (IQR)	Number of fraction/plan/GTV	Median (IQR)	Number of fraction/plan/GTV	*P*-value
*OTT with ET (min)*	All fractions	31 (20–39)	35	23 (20–31)	165	0.142
*Modulation factor (MU/Gy)*	All plans	269 (227–286)	32	338 (304–382)	182	≤10^−10^
*D*_*mean*_* BmP (Gy)*	All GTV volumes	1.4 (0.9–1.7)	32	0.9 (0.6–1.5)	179	0.038
	Total GTV volume ≤1 cc	0.35 (0.23–0.58)	6	0.33 (0.26–0.51)	43	0.819
	Total GTV volume 1 cc to 5 cc	1.06 (0.91–1.55)	10	0.84 (0.64–1.10)	64	0.059
	Total GTV volume >5 cc	1.57 (1.37–2.54)	16	1.70 (1.12–2.23)	72	0.607
*V*_*12Gy*_* BmP (cc)*	All GTV volume	7.5 (4.4–11.2)	32	5.0 (2.0–10.1)	179	0.058
	Total GTV volume ≤1 cc	1.80 (0.85–2.90)	6	1.20 (0–80–1.70)	43	0.278
	Total GTV volume 1 cc to 5 cc	6.00 (4.50–7.30)	10	4.40 (2.85–5.80)	64	0.081
	Total GTV volume >5 cc	11.15 (8.80–18.00)	16	11.6 (7.00–21.75)	72	0.812
*HI*_*RTOG*_* (relative value)*	All GTV volume	1.3 (1.3–1.4)	54	1.4 (1.3–1.5)	284	0.217
	Individual GTV volume ≤1 cc	1.37 (1.24–1.44)	24	1.40 (1.32–1.46)	123	0.171
	Individual GTV volume 1 cc to 5 cc	1.30 (1.27–1.38)	17	1.37 (1.30–1.41)	86	0.284
	Individual GTV volume >5 cc	1.29 (1.24–1.32)	13	1.27 (1.24–1.33)	75	0.545
*CI*_*Paddick*_* (relative value)*	All GTV	0.97 (0.96–0.98)	54	0.96 (0.94–0.96)	284	≤10^−9^
	Individual GTV volume ≤1 cc	0.96 (0.96–0.98)	24	0.96 (0.96–0.96)	123	0.011
	Individual GTV volume 1 cc to 5 cc	0.97 (0.96–0.98)	17	0.96 (0.94–0.96)	86	≤10^−3^
	Individual GTV volume >5 cc	0.98 (0.98–0.98)	13	0.94 (0.94–0.97)	75	≤10^−4^
*GI (relative value)*	All GTV	3.2 (2.9–4.0)	54	3.0 (2.7–3.5)	284	0.025
	Individual GTV volume ≤1 cc	3.93 (3.55–4.65)	24	3.44 (3.11–4.01)	123	0.002
	Individual GTV volume 1 cc to 5 cc	3.00 (2.90–3.21)	17	3.00 (2.81–3.23)	86	0.673
	Individual GTV volume >5 cc	2.67 (2.60–2.84)	13	2.57 (2.43–2.68)	75	0.094

As expected, median OTT were statistically significantly longer with use of the ET system compared to no ET (*p* < 0.00001). A statistically significant increase in the modulation factor (*p* = 0.002) was observed from the plans with 3 fractions of 9 Gy to the plans with 1 fraction of 13 Gy (Table 2).

Twenty-two tests were undertaken for the comparisons between the HD- and 120-MLC HA plans: four tests for each of D_mean_ and V_12_ _Gy_ of BmP, HI, CI, and GI index, one for the modulation factor (MU/Gy), and one for the OTT. For OTT there was no statistically significant difference between 120-MLC and HD-MLC HA treatments (*p* = 0.142). Statistically significant differences between HD- and 120-MLC were found for the GI for GTV volumes smaller than 1 cc (*p* = 0.002), and for the plan modulation factor which was higher for HD-MLC compared to 120-MLC (*p* < 10^−10^).

Statistically significant differences were found for the CI between the HD-MLC and 120-MLC, probably due to the large amount and the short range of the HD-MLC CI values; however, there are no clinically relevant differences in the observed values.

The HA plans created with 120-MLC and HD-MLC demonstrated comparable HI values. The median value of D_mean_ and V_12_ _Gy_ of BmP were lower for plans delivered with HD-MLC compared to the plan with 120-MLC. However, the difference was not statistically significant, possibly because of the limited amount of data gathered using 120-MLCs (Table 3).

### Quality assurance

The mean values (± one standard deviation) of the offset detected by the Winston–Lutz tests performed between September 2018 and June 2020 were 0.31 ± 0.07 mm and 0.32 ± 0.07 mm, while the maximum offset values were 0.53 ± 0.10 mm and 0.56 ± 0.12 mm for HD-MLC and 120-MLC, respectively [22]. The deviations were within the tolerance (0.75 mm average and 1 mm maximum) recommended by TG 142 and the ASTRO quality and safety guidelines for SRS [23, 24].

### Image-guidance and intrafractional motion

The analyses of the residual intrafractional positioning errors based on ET data of the first 100 patients treated with HD-MLC allow us to conclude that in 51% of the fractions, a couch adjustment was applied at the first couch rotation, in 20% at the second, and in 16% at the third couch rotation. No ET system-based adjustments were performed in 12% of the fractions.

**Fig. 2:**
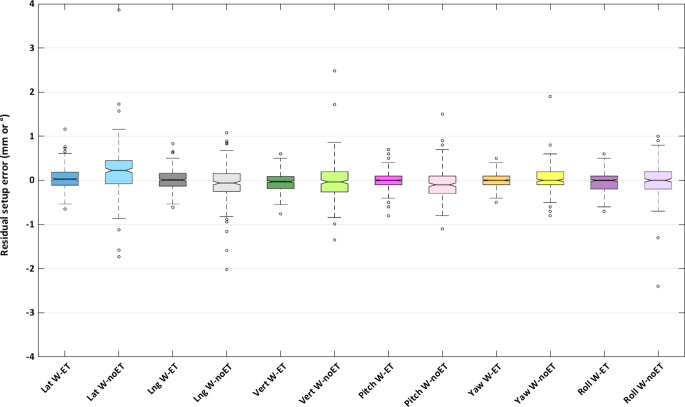
Residual intrafractional setup errors in lateral (*blue*), longitudinal (*grey*), and vertical (*green*) translations and pitch (*pink*), yaw (*yellow*), roll (*purple*) rotations, with W‑ET (*dark*) and W‑noET (*light*), respectively

The distributions of residual setup errors were, as expected, wider for W‑noET than for W‑ET (Fig. 2). In order to assess whether this is statistically significant, the distribution of distances from the median (Mdist) was calculated for each of the 12 distributions in Fig. 2, i.e., the absolute values of each setup error minus the median. The median of Mdist was statistically significantly higher for W‑noET compared to W‑ET for all the 6‑DOF of the couch (*p* < 10^−4^ for all translations and rotations).

**Fig. 3:**
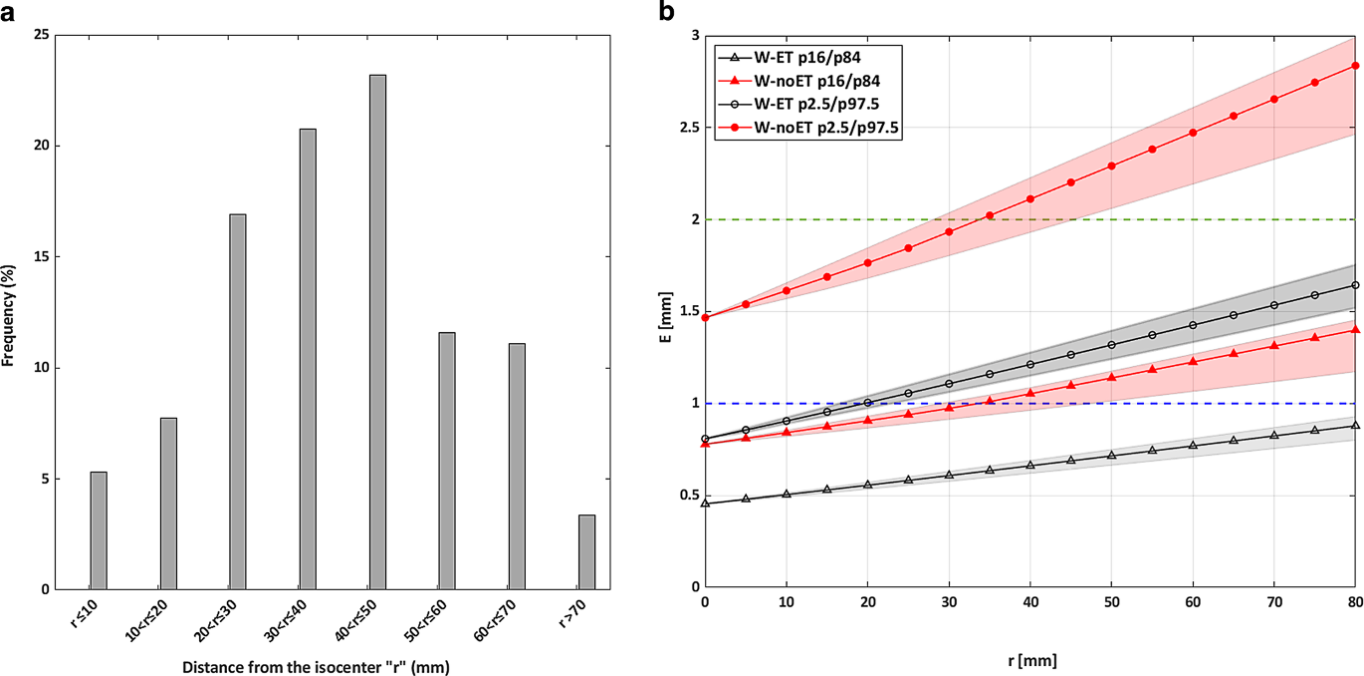
**a** Histogram of the distance “r” between the center of each single lesion and the plan isocenter for multi-BM treatment plans **b** Representation of the maximum deviation E (mm) as a function of the distance r (mm) for W‑ET (*grey*) and W‑noET (*red*) calculated for all combinations of residual intrafractional setup error of the 16th percentile (P_16_) to the 84th percentile (P_84_; *triangular markers*) and of the 2.5th (P_2.5_) percentile to the 97.5th percentile (p_97.5_; *circular markers*) for HD-MLC plans. The area covered by the six alternative r coordinate curves calculated for the vector E (described in Materials and methods), is illustrated as a *shaded area*. The *green* and *blue dotted lines* denote the 2‑mm and the 1‑mm GTV-PTV margin, respectively

The histogram displayed in Fig. 3a demonstrates, for the majority of the BMs, that the distance between the center of the BM and the plan isocenter was within 7 cm. Only 5 BM (3 patients) were situated more than 7.5 cm from the plan isocenter. 70% of the lesions for multi-BM treatment in this study were situated more than 3 cm from the isocenter.

The maximum displacement vector (E) of the center of the BM for a given rotational setup error increases as the distance (r) between the plan isocenter and the center of the BM increases. E likewise depends on the magnitude of the residual setup errors (Fig. 3b). For 95% of single lesions or BM within 2 cm of the plan isocenter, the net deviation of the BM stayed within the 1‑mm margin for W‑ET. For a distance between the plan isocenter and the center of the lesion of more than 3.5 cm, only 68% of the BMs had a net deviation of the BM within the 2‑mm margin with W‑noET while 95% of the targets stayed within the 2‑mm with W‑ET.

### Dosimetric effects of rotational errors

**Fig. 4:**
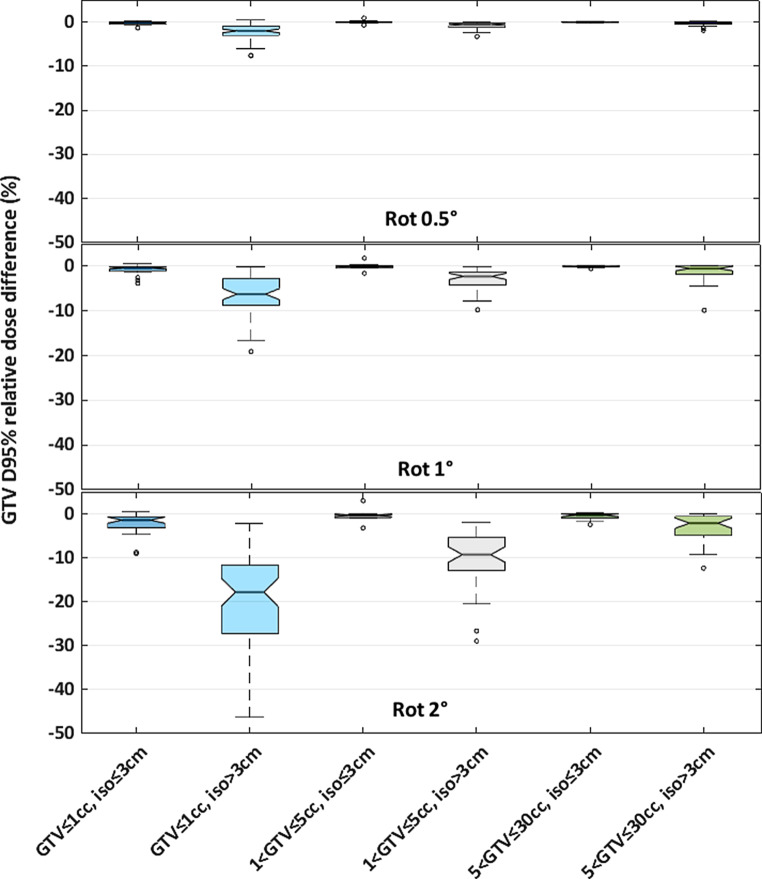
Relative dose error boxplot of GTV D_95%_ (dose received by 95% of the GTV volume) for three GTV volume groups: ≤1 cc (*blue*), 1 to 5 cc (*grey*), 5 to 30 cc (*green*); and for two distances between the center of the BM and the plan isocenter: ≤3 cm (*dark color*) and >3 cm (*light color*) for rotational errors of 0.5°, 1°, and 2°

When rotational errors were small (0.5° of rotation), all BMs were well covered, with a maximal median (IQR) decrease in GTV D_95%_ of 2.0% (0.9 to 3.0%). If the center of the BM was situated within 3 cm from the plan isocenter, all the simulated rotational errors had a minor impact on BM coverage, with a maximum median decrease in GTV D_95%_ for all the BM (from 0 to 30 cc) of 1.4% (0.7 to 3.1%; Fig. 4).

When the distance between the center of the BM and the plan isocenter was >3 cm, the large BMs (5 to 30 cc) were less sensitive to the rotational error than the small BM (0 to 1 cc). For example, for a rotational error of 1°, the median (IQR) of the decrease in GTV D_95%_ for the small BM (0 to 1 cc) was 6.3% (2.8 to 8.8%) while the value for larger BM (5 to 30 cc) it was 0.6% (0.0 to 1.8%).

The analysis of the data indicated that the rotational error had limited impact on the dose delivered to the OAR and to the BmP (results not reported).

## Discussion

This study summarizes data from 202 patients treated with the HA solution, with both 120-MLC and HD-MLC, and encompasses, to our knowledge, more data than previously published HA studies which included only 15 to 30 patients [12, 14, 15].

### 120-MLC vs. HD-MLC

As described previously, a CI close to one is a predictor of local control and sparing of the OAR, while an increase of GI to above three can lead to a higher V_12_ _Gy_ to the BmP, which is related to the risk of radionecrosis [15, 16]. The GI median (IQR) value in our study was higher for small BMs (<1 cc) for plans delivered with 120-MLC (GI = 3.93 [3.55–4.65]) compared to the ones with HD-MLC (GI = 3.44 [3.11–4.01]), while no statistically significant differences were observed for GTV larger than 1 cc. Similar to our study, Ruggieri et al. [12] have reported a GI mean ± SD value of 4.4 ± 1.2 for treatment delivered with 120-MLC (BM volumes of 0.5 cc to 27.9 cc), which was higher than the value observed by Ohira et al. [25] where the GI mean ± SD value was 3.1 ± 0.4 for treatment delivered with HD-MLC (BM size range from 0.5 to 15.6 cc). However, we did not find a statistically significant difference for GI between the two MLCs (*p* = 0.025) after correction for multiple testing. For all our plans, the V_12_ _Gy_ of BmP was within the recommendation of 10 cc [12] and the results indicate that the HD-MLC was better at sparing normal brain tissue compared to the 120-MLC, although the results were not statistically significant. Further investigation into this could be of value, e.g., a dose plan study with plans for both MLCs for the same patients. This was, however, outside the scope of this work.

The CI, HI, and OTT values reported by Ruggieri et al. [12] and Ohira et al. [25] were comparable to the data found in our study.

The 22 tests carried out are not independent and, thus, the Bonferroni correction for multiple testing likely overcorrects if the goal is to keep the overall alpha at 0.05. For that reason, we reported all *p*-values, making it easy to see what the consequence of the correction is.

### W-noET vs. W-ET

With high dose per fraction and only one to three fractions, SRS treatments are more sensitive to random setup errors compared to conventional radiotherapy. Therefore, it is particularly important to localize the target with high precision using a solid IGRT workflow and machine control check. As explained in this study, the control check was implemented as recommended by ASTRO to prevent error in the SRS treatment at each step of the workflow [16]. The results of Winston–Lutz tests performed over 2 years were similar to those of the study by Gao et al. [19], where a mean offset of 0.32 mm and a maximum offset of 0.52 mm were observed. Different methods to perform the Winston–Lutz test were used in the literature, as was well described by Rowshanfarzad et al. [20]. In our study, the mimicking of the real clinical situation using weight placed on the couch as mentioned by Gao et al. [19] was not used. Moreover, further investigations have to be done in order to use rotation-induced couch shift MPC control as a substitute for the Winston–Lutz test as described by Barnes et al. and Clivio et al. [26, 27].

Treating multi-BM with a one isocenter plan requires proper quantification of the geometric uncertainty of the patient position for the specific immobilization setup device and treatment positioning system used. In our study, the maximum deviations were ±1.2 mm (LAT), ±0.8 mm (CC), ±0.6 mm (AP), ±0.8° (pitch), ±0.5° (yaw), ±0.7° (roll) for HD-MLC treatment with W‑ET, which was comparable with the results of the literature [28, 29]. Based on our institution’s data, it is possible to use W‑noET for the treatment of targets within 3 cm from the plan isocenter when applying a 2-mm GTV–PTV margin.

Moreover, the OTT was decreased by 50% by excluding the ET images. However, the setup errors may be underestimated for W‑noET, as the residual setup errors were not recorded after the first couch adjustment based on the ET system for this specific workflow.

For treatment of multi-BM situated more than 3 cm from the plan isocenter, which encompass 70% of the BMs in case of multi-BM treatment (Fig. 3a), dose coverage was only ensured by W‑ET with a GTV–PTV margin of 2 mm. The results of our study suggest the possibility of incorporating different GTV–PTV margins in the treatment planning process, according to the distance from the plan isocenter and to the volume of each lesion. For example, the GTV–PTV margin could be reduced to 1 mm for lesions within 3 cm from the plan isocenter without compromising BM coverage for W‑ET.

### Compromised target coverage due to residual isocenter rotational errors

For rotational errors of 1° and for the small targets (≤1 cc) situated more than 3 cm from the plan isocenter, the median (IQR) decrease in GTV D_95%_ was 6.3% (2.8 to 8.8%) with a 2-mm GTV–PTV margin. Roper et al. have observed similar results and concluded that target coverage worsened with increasing rotational error, distance to the plan isocenter, and decreasing PTV volume [30]. This result underlines the importance of using W‑ET for targets situated more than 3 cm from the plan isocenter to verify the patient’s positioning with accuracy and avoid GTV underdosage.

### Limitations

Some limitations in this study should be considered. It was a retrospective study. The number of plans utilizing 120-MLC (*n* = 32) was limited compared to the ones with HD-MLC (*n* = 170). The intrafractional patient error was based on only one IGRT system (ET) for which the accuracy was not evaluated in this study. However, it was previously evaluated by Ackerly et al. [31] and Li et al. [32]. Finally, the relative dose error due to rotation of the patient was not re-calculated in the TPS used for SRS plan creation, but rather based on a simple geometrical shift of the dose distribution.

## Conclusion

We have used both MLCs (5- and 2.5-mm central leaf width) to treat SRS BMs with good plan quality; however, this study indicates that the HD-MLC (2.5-mm central leaf width) is better at sparing the healthy brain. For BM within 3 cm of the plan isocenter, the initial patient IGRT setup (W-noET) is sufficient to ensure BM coverage when a 2-mm margin is applied. The GTV–PTV margin could be reduced to 1 mm for lesions within 2 cm of the plan isocenter without compromising BM coverage for W‑ET. For BM situated far from the plan isocenter (>3 cm) when using a 2-mm margin or for all BM when using a 1-mm margin, our study suggests that patient positioning errors at each couch rotation should be corrected (W-ET) to avoid compromising BM coverage.

## Abbreviations

AP: Anteroposterior
BM: Brain metastases
BmP: Brain minus planning target volume
CBCT: Cone beam computed tomography
CC: Craniocaudal
CI: Conformity index
D_max_: Maximum dose
D_mean_: Mean dose
D_p_: Prescribed dose
dpi: Dot per inch
DOF: Degree of freedom
E: Magnitude of the maximum displacement vector of the center of the target
ET: ExacTrac
EPID: Electronic portal imaging device
GI: Gradient index
GK: Gamma Knife
GTV: Gross tumor volume
HA: HyperArc
HD: High definition
HI: Homogeneity index
IGRT: Image-guided radiotherapy
IQR: Interquartile range
LAT: Lateral
Linac: Linear accelerator
M: Median value
MLC: Multileaf collimator
MRI: Magnetic resonance imaging
MU: Monitor unit
NTO: Normal tissue objective
OAR: Organ at risk
OBI: On-board imaging
OTT: Overall treatment time
PCT: Planning computed tomography
PTV: Planning target volume
PV: Prescription isodose volume
QA: Quality assurance
r: Distance between the center of the target and the mechanical isocenter
Rpitch, Rroll, Ryaw: Rotation matrix for pitch, roll, and raw direction
RGB: Red/green/blue
SAD: Source–axis distance
SRS: Stereotactic radiosurgery
T: Translation matrix
TV: Target volume
VDp: Volume of the target covered by the prescribed dose
VMAT: Volumetric modulated arc therapy
V_12Gy_: Volume receiving at least 12 Gy
WBRT: Whole-brain radiotherapy
